# [Corrigendum] Protective effects of atorvastatin on cerebral vessel autoregulation in an experimental rabbit model of subarachnoid hemorrhage

**DOI:** 10.3892/mmr.2025.13673

**Published:** 2025-09-03

**Authors:** Jun-Hui Chen, Ting Wu, Li-Kun Yang, Lei Chen, Jie Zhu, Pei-Pei Li, Xu Hu, Yu-Hai Wang

Mol Med Rep 17: 1651–1659, 2018; DOI: 10.3892/mmr.2017.8074

Following the publication of the above paper, an interested reader drew the authors’ attention to the fact that images showing TUNEL staining of hippocampus following SAH induction in Fig. 2A, and the western blot data in Fig. 7, apparently had already been previously published in an article written by the same research group in the journal *International Journal of Molecular Medicine*. After asking the authors to provide an explanation for the inclusion of the same data in the above paper, the authors responded to say that these two studies were finished at around the same time in their laboratory, both of which explored the neuroprotective effects of atorvastatin following subarachnoid hemorrhage. The study published in *International Journal of Molecular Medicine* focused on the effect of atorvastatin on AQP4 expression and early brain injury following subarachnoid hemorrhage, whereas the above study explored the protective effects of atorvastatin on cerebral vessel autoregulation and early brain injury after subarachnoid hemorrhage. As the results of the apoptosis experiments were applicable to both studies, the authors deemed it appropriate to include the same data and figures (which were the TUNEL staining data in Fig. 4 and the caspase-3 western data in Fig. 7 in the above paper) in the two studies.

In response to the identification of the matching data in these two papers that were published by this research group, the authors have submitted revised versions of Figs. 4 and 7, now featuring data from the repeated experiments in either case. The Editor of *Molecular Medicine Reports* has considered that it would be prudent to publish a corrigendum for the above paper featuring these alternative data, and the revised versions of Figs. 4 and 7 are presented on the next page. The authors are grateful to the Editor for allowing them the opportunity to publish this corrigendum for the purposes of the scientific record.

## Figures and Tables

**Figure 4.**
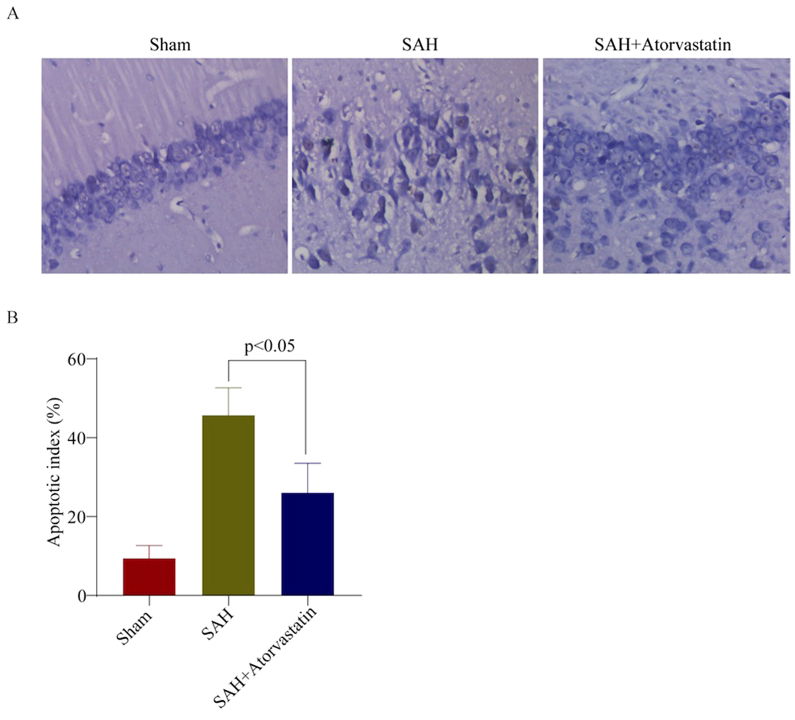
TUNEL staining of hippocampus following SAH induction. (A) Few apoptotic cells were observed in Sham group rabbits. TUNEL-positive staining was markedly increased in SAH rabbits, which was notably reduced by atorvastatin treatment. (B) Quantitative analysis demonstrated a significant increase in the number of apoptotic cells (number/mm^2^) by SAH induction compared with the number of apoptotic cells in the Sham group; *P<0.05. A significant reduction in number of apoptotic-positive cells was observed in the SAH + atorvastatin group compared with the SAH group; ^#^P<0.05. SAH, subarachnoid hemorrhage; TUNEL, terminal deoxynucleotidyl-transferase-mediated dUTP nick-end labeling.

**Figure 7.**
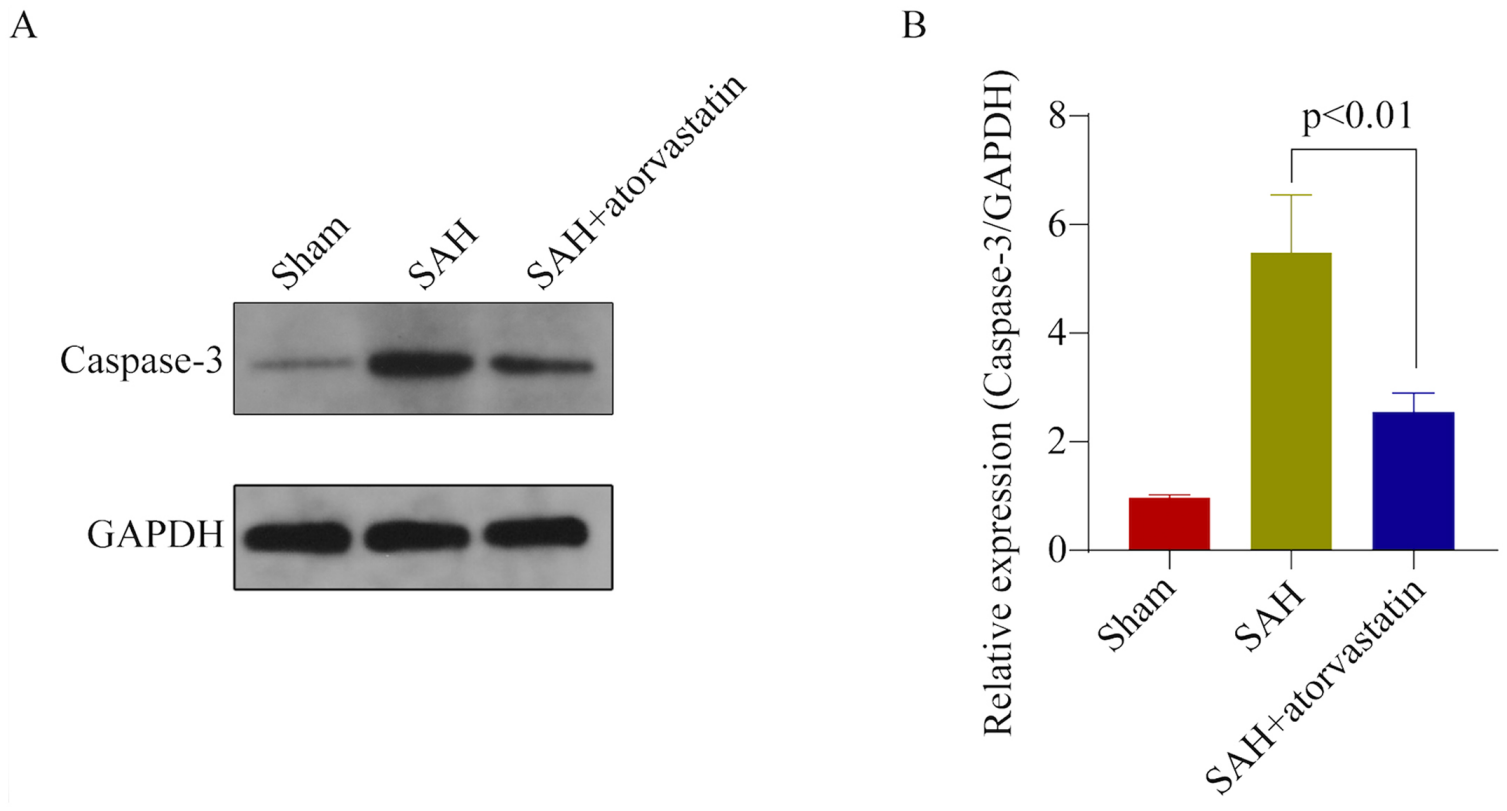
Caspase-3 protein expression in the hippocampus. (A) Representative western blot of Caspase-3 protein expression. (B) Densitometric analysis of Caspase-3 protein expression. Data are presented as the mean ± standard deviation. Caspase-3 protein expression in the Sham group was significantly lower than SAH group and SAH + atorvastatin group (n=8/group, ^#^P<0.01). Caspase-3 expression levels were significantly decreased in the SAH + atorvastatin group vs. the SAH group; *P<0.05. Equal protein loading was confirmed by intracellular GAPDH protein expression. SAH, subarachnoid hemorrhage.

